# Burden of rare coding variants reveals genetic heterogeneity between obese and non-obese asthma patients in the African American population

**DOI:** 10.1186/s12931-022-02039-0

**Published:** 2022-05-06

**Authors:** Yichuan Liu, Hui-Qi Qu, Jingchun Qu, Xiao Chang, Frank D. Mentch, Kenny Nguyen, Lifeng Tian, Joseph Glessner, Patrick M. A. Sleiman, Hakon Hakonarson

**Affiliations:** 1 Center for Applied Genomics, Children’s Hospital of Philadelphia, Philadelphia, PA 19104 USA; 2 Department of Pediatrics, The Perelman School of Medicine, University of Pennsylvania, Philadelphia, PA 19104 USA; 3 Division of Human Genetics, Children’s Hospital of Philadelphia, Philadelphia, PA 19104 USA; 4 Division of Pulmonary Medicine, Children’s Hospital of Philadelphia, Philadelphia, PA 19104 USA; 5 Faculty of Medicine, University of Iceland, 101 Reykjavik, Iceland

**Keywords:** Asthma, Obesity, Comorbidity, Rare coding variants, Whole genome sequencing (WGS)

## Abstract

**Background:**

Asthma is a complex condition largely attributed to the interactions among genes and environments as a heterogeneous phenotype. Obesity is significantly associated with asthma development, and genetic studies on obese vs. non-obese asthma are warranted.

**Methods:**

To investigate asthma in the minority African American (AA) population with or without obesity, we performed a whole genome sequencing (WGS) study on blood-derived DNA of 4289 AA individuals, included 2226 asthma patients (1364 with obesity and 862 without obesity) and 2006 controls without asthma. The burden analysis of functional rare coding variants was performed by comparing asthma vs. controls and by stratified analysis of obese vs. non-obese asthma, respectively.

**Results:**

Among the top 66 genes with P < 0.01 in the asthma vs. control analysis, stratified analysis by obesity showed inverse correlation of natural logarithm (LN) of *P* value between obese and non-obese asthma (r = − 0.757, P = 1.90E−13). Five genes previously reported in a genome-wide association study (GWAS) on asthma, including *TSLP*, *SLC9A4*, *PSMB8*, *IGSF5*, and *IKZF4* were demonstrated association in the asthma vs. control analysis. The associations of *IKZF4* and *IGSF5* are only associated with obese asthma; and the association of *SLC9A4* is only observed in non-obese asthma. In addition, the association of *RSPH3* (the gene is related to primary ciliary dyskinesia) is observed in non-obese asthma.

**Conclusions:**

These findings highlight genetic heterogeneity between obese and non-obese asthma in patients of AA ancestry.

**Supplementary Information:**

The online version contains supplementary material available at 10.1186/s12931-022-02039-0.

## Background

Asthma is a chronic respiratory disease which affects more than 20 million people in United States [1]. While significant progress has been made in the field of asthma genetics in the past decade, the clinical implications of genetic loci reported in previous studies remain unclear due to heterogeneous phenotypes and complex gene-environment interactions [2]. Obesity, the most common type of metabolic health problems, has been suggested as both a major risk factor and a disease modifier for asthma in both children and adults [3, 4]. Given the important contributions of rare coding variants in asthma [5], a genetic study focusing on the impact of rare coding variants in asthma patients with or without obesity is warranted.

Moreover, asthma research is significantly lacking in minority populations, such as African Americans (AA). This is particularly important given the fact that individuals of AA ancestry are 42% more likely to be affected by asthma, compared to European Americans (EA), based on demographics data from the American Lung Association (https://www.lung.org/research/trends-in-lung-disease/asthma-trends-brief/current-demographics). To address the key missing piece of the puzzle, we performed burden analysis of rare coding variants in 4289 AA individuals, including 1364 asthma patients with obesity and 862 asthma patients without obesity, in comparison with 2006 controls, to explore the genetic differences in asthma patients with or without obesity.

## Methods

### Patient cohorts

**Fig. 1:**
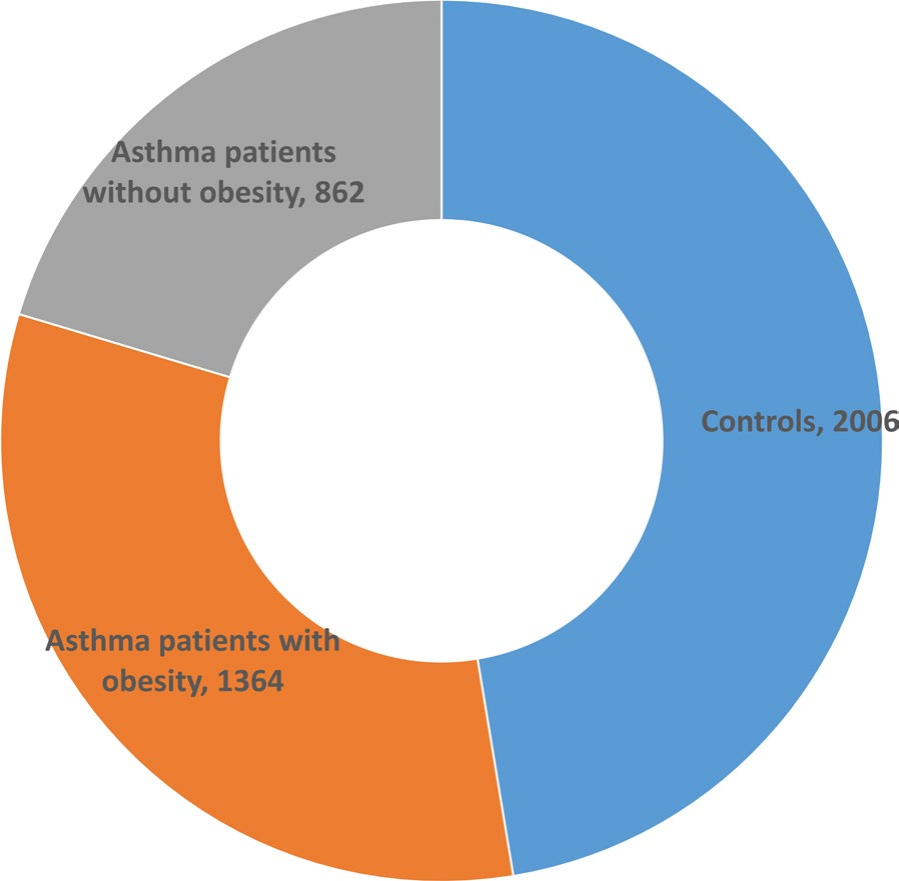
Pie chart for the phenotype groups

**Fig. 2:**
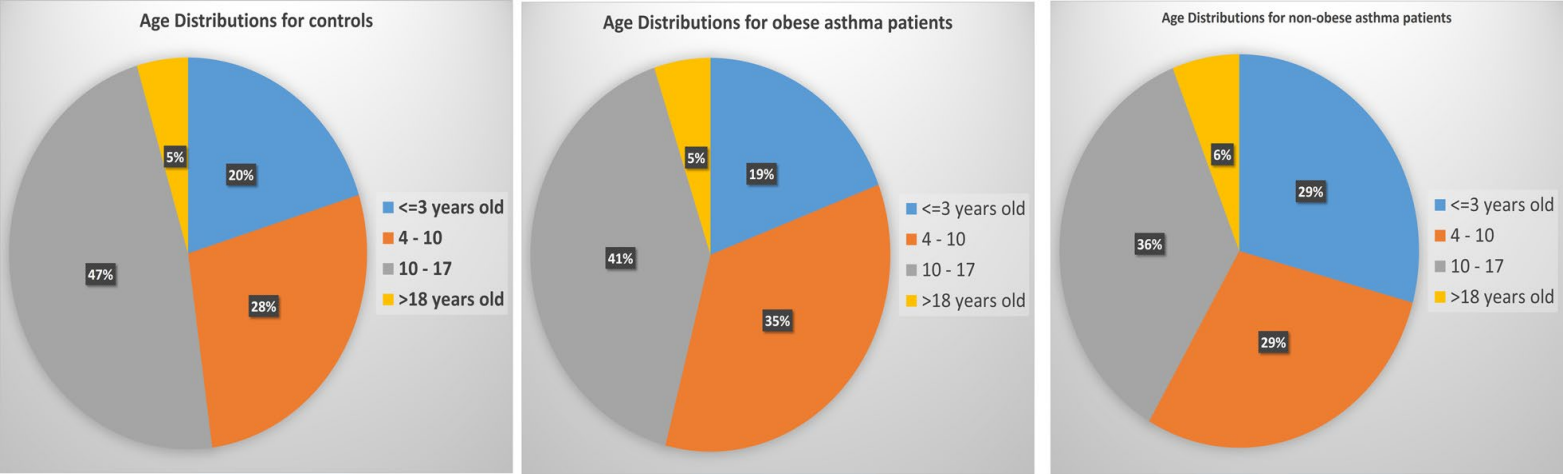
Age distributions of controls, obese asthma patients, and non-obese asthma patients

The patients selected in this study are from the Center for Applied Genomics (CAG) at The Children’s Hospital of Philadelphia (CHOP). WGS were generated through the NHLBI Trans Omics for Precision Medicine (TOPMed) WGS Program (https://www.ncbi.nlm.nih.gov/projects/gap/cgi-bin/study.cgi?study_id=phs001661.v2.p1). All the 4289 AA patients were selected from the CAG biobank, including 2226 patients with a diagnosis of asthma. In this study, 1364 asthma patients (680 females and 684 males) were diagnosed with obesity, and 862 asthma patients (364 females and 498 males) were without obesity in comparison with 2006 control subjects (1014 females and 992 males) (Fig. 1). The age distributions of the three groups are shown in Fig. 2. The patients were approached during regular hospital visits at multiple clinics, including emergency room, ambulatory settings, general pediatrics, and specialty pediatric practices, including pulmonary and allergy clinics. The patients were in the age range of 5–21 years and receiving healthcare at CHOP. Parental consent was obtained for individuals under 18 years old and assent was also obtained for subjects aged 7–17 years. The consent allowed samples to be analyzed using the genomic technologies herein, to address the research questions proposed. Parents had the option to opt-in to permit regular updates of their child's electronic health record data (EHR) and to be re-contacted for future study, which essentially everyone did.

### Electronic health record (EHR) data extractions

The Center for Applied Genomics (CAG) at CHOP maintains a de-identified extract of clinical data from the CHOP EHR database for consented patients. This database contains longitudinal information about visits, diagnoses, medical history, prescriptions, procedures, and lab tests.

### Whole genome sequencing (WGS) data processing and variation detection

The WGS data were generated by the TOPMED project. As described in the TOPMED project, DNA was isolated from whole blood, while DNA quantity and sex discordance have been checked in the quality assessments. Libraries for WGS were created using the Illumina’s TruSeq DNA PCR-Free Library Preparation Kit. Whole Genome Sequencing was performed on the Illumina HiSeq X Ten platform with paired end 150 bp reads. The bcl2fastq v2 15.0 package was used to generate individual FASTQ files. The alignment pipeline can be found at https://github.com/CCDG/Pipeline-Standardization/blob/master/PipelineStandard.md. Mutation burden analysis was based on the variant call format (VCF) files. Common variants with minor allele frequency (MAF) greater than 0.001 in the African population based on the Exome Aggregation Consortium (ExAC) database [6] were removed.

### Mutation burden analysis

Functional candidate variants were selected by the prediction results with at least one genetic variant prediction software, i.e. SIFT_pred = "D" or Polyphen2_HDIV_pred = "D" or Polyphen2_HDIV_pred = "P" or Polyphen2_HVAR_pred = "D" or Polyphen2_HVAR_pred = "P" or LRT_pred = "D" or MutationTaster_pred = "A" or MutationTaster_pred = "D" or MutationAssessor_pred = "H" or MutationAssessor_pred = "M" or FATHMM_pred = "D" or PROVEAN_pred = "D" or MetaSVM_pred = "D" or MetaLR_pred = "D", annotated by the ANNOVAR software [7]. Functional variants in cases and controls were counted with the Test Rare vAriants with Public Data (TRAPD) software [8]. Gene-wide burden analysis of the functional variants in the cases was done by one tailed Fisher exact test, compared with that in the controls in the dominant inheritance model. A significant *P* value means increased mutation burden in the cases (or the former group), compared to the controls (or the latter group). Candidate genes with p value less than 0.01 in the mutation burden analysis comparing asthma cases vs controls were selected for further analysis.

## Results

**Fig. 3:**
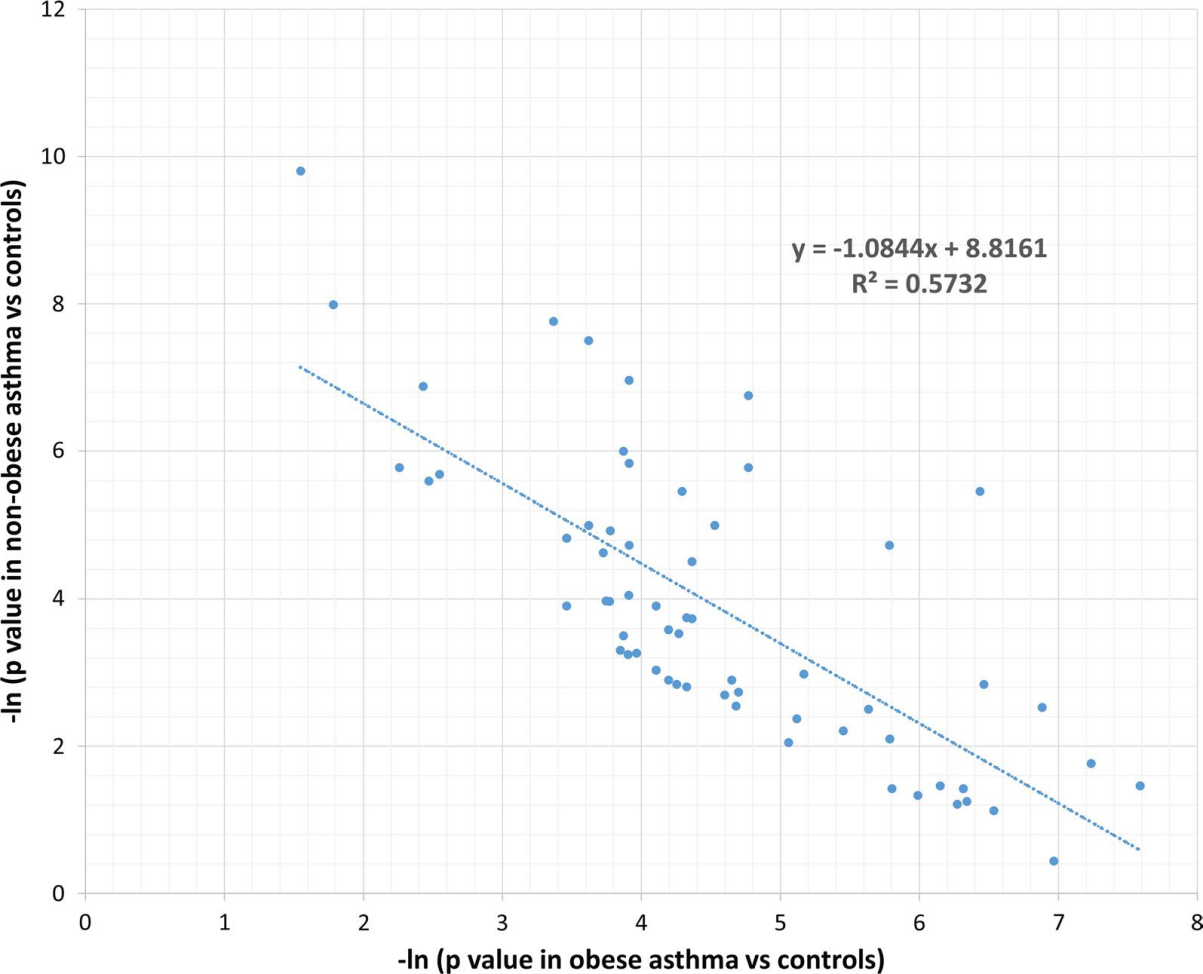
Negative correlations of 66 genes identified by mutation burden tests between obese asthma and non-obese asthma. X-axis is the − ln (P value) of the obese asthma (vs. controls) tests and Y-axis is the − ln (P value) of the non-obese asthma (vs. controls) tests

Based on the mutation burden analysis, 66 genes have *P* value less than 0.01 by comparing asthma cases *vs.* non-asthma controls (Additional file 1: Table S1). Stratified analysis based on the 66 genes by the comorbidity of obesity showed inverse correlation of the natural logarithm (LN) of *P* value between the two subgroups (Fig. 3). In other words, − LN(*P* value)s of these 66 genes in obese asthma vs. controls are negatively correlated with those in non-obese asthma vs. controls (r = − 0.757, *P* = 1.90E−13). Six genes (Table 1) have been reported of genetic association with asthma susceptibility by previous large-sample GWASs (Additional file 2: Table S2). In addition, the gene *RSPH3* causing primary ciliary dyskinesia is also further examined. With the Bonferroni corrected significant level of α = 0.05/7 = 7.14E−03, 5 of the 6 genes remain significant after correction for multiple testing. Consequently, stratified analysis based on the comorbidity of obesity suggested potential heterogeneous effects from 3 of the 5 genes, i.e., with α = 7.14E−03, the associations of *IKZF4* and *IGSF5* are only seen in obese asthma; and the associations of *RSPH3* and *SLC9A4* are only seen in non-obese asthma. These findings suggest the genetic heterogeneity between obese vs. non-obese asthma.

**Table 1:** Mutation burden analysis of 6 genes identified in previous GWAS studies

Gene ID	*P* value (asthma vs controls)	*P* value (obesity asthma vs controls)	*P* value (non-obese asthma vs controls)	*P* value (obese asthma vs non-obese asthma)	*P* value (non-obese asthma vs obese asthma)	*P* value (obesity)*
*SLC9A4*	0.005	0.088	1.03E−03	0.976	0.056	0.273
*TSLP*	1.43E−03	0.027	5.50E−04	0.964	0.123	0.149
*PSMB8*	0.005	0.013	0.024	0.629	0.549	0.577
*GPR182*	0.010	0.013	0.061	0.519	0.711	0.687
*IGSF5*	0.007	1.89E-03	0.298	0.074	0.970	0.420
*IKZF4*	0.009	9.40E-04	0.643	0.015	0.997	0.017
*RSPH3*	7.87E−03	0.168	3.40E−04	0.993	0.009	0.010

*Logistic regression adjusted for asthma

## Discussion

Asthma is a complex disorder with a heterogeneous phenotype, and previous studies have shown that asthma patients with obesity do not respond in the same way to medications as patients without obesity. Beside environmental factors, genetic susceptibility to asthma is associated with multiple genetic factors [9]. As a result, exploration of the genetic differences of asthma patients with or without obesity would be critical to decipher the medication responses at the molecular level, in order to guide a more precise treatment.

In a total of 66 genes with *P* < 0.01 in the comparison of asthma cases vs. controls, we observed strongly negative correlations in the stratification analysis by the status of obesity (r = − 0.757, P = 1.90E−13). This finding highlights the genetic heterogeneity between obese asthma and non-obese asthma. Limited by the sample size of our study, none of these genes acquired genome-wide significance. Therefore, we focused on 6 of the 66 genes that were previously identified and shown to be associated with asthma by large scale GWASs. Five of these 6 genes remain significant after correction for multiple testing. Three of the genes delineated clear heterogeneous effects between obese asthma and non-obese asthma, with *IKZF4* and *IGSF5* showing association with obese asthma, and *SLC9A4* with non-obese asthma.

The IKAROS family zinc finger 4 gene (*IKZF4*) encodes a transcription factor, controlling the function of FOXP3-positive regulatory T cells [10]. Genetic variation of *IKZF4* have been reported of association with both asthma in both European [11] and East Asian [12]. Meanwhile, the frequency of the risk allele of the asthma associated SNP rs1701704 is significantly lower in African population compared to other populations [13], while our study suggests additional risk from rare coding variants. The immunoglobulin superfamily member 5 gene (*IGSF5*) encodes a member of the immunoglobulin superfamily and provides adhesion at cell–cell tight junctions [14]. Regarding its association with asthma [15], coding variants of *IGSF5* have been shown of correlation with inflammatory cell infiltration due to impaired adhesion function, and *IGSF5* has been reported in the GWAS of coronary heart disease in the African American population [16]. In contrast to the associations of *IKZF4* and *IGSF5* seen in obese asthma, the genetic association of *SLC9A4*, encoded by the solute carrier family 9 member A4 gene, is only seen in non-obese asthma. In addition to its association with asthma, it has also been shown to be associated with a number of pediatric autoimmune diseases [17].

As shown by a recent study [18], as well as our own Mendelian randomization analysis (unpublished data), obesity is a causal factor of asthma. In our study, in addition to the associations with asthma, the gene *IKZF4* showed nominal significance with obesity in the logistic regression analysis adjusted for asthma, implying *IKZF4* variants might contribute to asthma through obesity.

## Conclusions

In conclusion, we conducted a systemic burden analysis of rare coding variants of functional interest in asthma patients of AA ancestry and compared obese asthma with non-obese asthma patients. The results showed that 5 genes previously reported by GWAS studies on asthma are enriched for rare coding variants in asthma patients. In addition, genetic heterogeneity was suggested by stratified analysis on comorbid obesity. Two obese asthma genes and one non-obese asthma gene were highlighted. However, the sample size presents a limitation of this study as its statistical power is limited. The asthma genes and their specific effects in obese vs. nonobese asthma need verification in independent samples. In addition, other loci suggested of specific effects in the stratified analysis also warrant for further investigation. For example, the radial spoke head 3 gene (*RSPH3*) is only associated with non-obese asthma. As a plausible functional candidate, *RSPH3* encodes a protein kinase A anchoring protein [19]. *RSPH3* mutations cause primary ciliary dyskinesia [20]. This gene may thus imply ciliary dysfunction in non-obese asthma.

## Supplementary Information

**Additional file 1:** Mutation burden analysis outputs of 66 genes that have P value less than 0.01 by comparing asthma cases vs. non-asthma controls.

**Additional file 2:** Six genes have been reported of genetic association with asthma susceptibility by previous GWASs.

## Data Availability

The data has been uploaded to the database of Genotypes and Phenotypes (dbGaP, https://www.ncbi.nlm.nih.gov/gap/) with the accession number phs001661.v2.p1.
